# MicroRNA-26a supports mammalian axon regeneration *in vivo* by suppressing GSK3*β* expression

**DOI:** 10.1038/cddis.2015.239

**Published:** 2015-08-27

**Authors:** J-J Jiang, C-M Liu, B-Y Zhang, X-W Wang, M Zhang, Saijilafu, S-R Zhang, P Hall, Y-W Hu, F-Q Zhou

**Affiliations:** 1 Department of Anesthesiology, Shengjing Hospital of China Medical University, Shenyang, Liaoning 110004, People's Republic of China; 2 Department of Orthopaedic Surgery and Neuroscience, The Johns Hopkins University School of Medicine, Baltimore, MD 21287, USA; 3 State Key Laboratory of Reproductive Biology, Institute of Zoology, Chinese Academy of Sciences, Beijing 100190, People's Repubic of China; 4 Department of Orthopaedics, the First Affiliated Hospital, Orthopaedic Institute, Soochow University, Suzhou 215123, Jiangsu, People's Republic of China; 5 The Solomon H Snyder Department of Neuroscience, The Johns Hopkins University School of Medicine, Baltimore 21287, MD, USA

## Abstract

MicroRNAs are emerging to be important epigenetic factors that control axon regeneration. Here, we report that microRNA-26a (miR-26a) is a physiological regulator of mammalian axon regeneration *in vivo*. We demonstrated that endogenous miR-26a acted to target specifically glycogen synthase kinase 3*β* (GSK3*β*) in adult mouse sensory neurons *in vitro* and *in vivo*. Inhibition of endogenous miR-26a in sensory neurons impaired axon regeneration *in vitro* and *in vivo*. Moreover, the regulatory effect of miR-26a was mediated by increased expression of GSK3*β* because downregulation or pharmacological inhibition of GSK3*β* fully rescued axon regeneration. Our results also suggested that the miR-26a-GSK3*β* pathway regulated axon regeneration at the neuronal soma by controlling gene expression. We provided biochemical and functional evidences that the regeneration-associated transcription factor Smad1 acted downstream of miR-26a and GSK3*β* to control sensory axon regeneration. Our study reveals a novel miR-26a-GSK3*β*-Smad1 signaling pathway in the regulation of mammalian axon regeneration. Moreover, we provide the first evidence that, in addition to inhibition of GSK3*β* kinase activity, maintaining a lower protein level of GSK3*β* in neurons by the microRNA is necessary for efficient axon regeneration.

Successful axon regeneration requires the neurons to have high intrinsic axon growth ability, which is controlled by the expression of many regeneration-associated genes in the neuronal soma.^1^ The inability of neurons in the mature mammalian central nervous system (CNS) to regenerate their axons is largely because of the loss of intrinsic axon growth ability.^1, 2^ As a result, modulation of gene expression has generated, to date, the most robust axon regeneration in the CNS.^3^ However, our understanding of how gene expression is controlled after axon injury remains very limited.

Epigenetic regulation independent of changes to DNA sequences is emerging to be a key cellular mechanism to control gene expression, in particular in proliferating cells, such as cancer and stem cells. We know much less about the roles of epigenetic modification in postmitotic neurons during axon growth and regeneration. Several recent studies have investigated the roles of microRNAs in the regulation of axon regeneration. For instance, in animals lacking the Dicer protein, which is crucial for microRNA processing,^4^ sensory axon regeneration was impaired, suggesting an important role of microRNAs. Indeed, a few profiling studies have reported that the expression levels of many microRNAs are changed in adult mouse sensory neurons after the peripheral nerve injury.^5, 6, 7^ However, to date, very few studies have examined the roles of microRNAs in the regulation of mammalian axon regeneration *in vivo*. Our recent study^8^ has provided the first *in vivo* evidence that microRNA-138 and its target histone deacetylase SIRT1 have important roles in the regulation of gene expression during mammalian axon regeneration *in vivo*.

Many previous studies, including ours, have shown that glycogen synthase kinase 3 (GSK3) signaling has very important roles in the regulation of axon regeneration. For instance, we have shown that moderate inactivation of GSK3 at the nerve growth cone is necessary for efficient axon regeneration by promoting microtubule stability.^9^ In addition, our recent study has also revealed that localized inactivation of GSK3 at the neuronal soma is required for axon regeneration by controlling nerve injury-induced gene expression.^10^ Moreover, either pharmacological inhibition of GSK3^11^ or conditionally knocking out GSK3*β*^12^ is able to promote axon regeneration in the spinal cord. However, to date, all previous studies of GSK3 have focused on the regulation of its kinase activity and there is no report about whether GSK3 expression is regulated during axon regeneration.

In this study, we have reported that microRNA-26a (miR-26a) functions to regulate mammalian axon regeneration *in vivo*. Inhibition of endogenous miR-26a in adult mouse sensory neurons led to markedly impaired axon regeneration *in vitro* and *in vivo*. Moreover, the regulatory effect of miR-26a on axon regeneration was mediated by targeting and controlling the expression of GSK3*β*. The knocking down of GSK3*β* fully rescued axon regeneration impaired by miR-26a inhibition. Moreover, we provided evidence that the miR-26a-GSK3*β* pathway regulated axon regeneration through controlling the expression of transcription factor Smad1, a well-known regeneration-associated protein.^10, 13^ Collectively, our study identified a novel miR-26a-GSK3*β*-Smad1 pathway in the regulation of axon regeneration-associated gene expression. In addition, we have revealed a new regulatory mechanism of GSK3 signaling: a lower level of GSK3 protein needs to be maintained by microRNAs in neurons to support efficient axon regeneration.

## Results

### miR-26a targets GSK3*β* in adult mouse sensory neurons *in vitro* and *in vivo*

**Figure 1:**
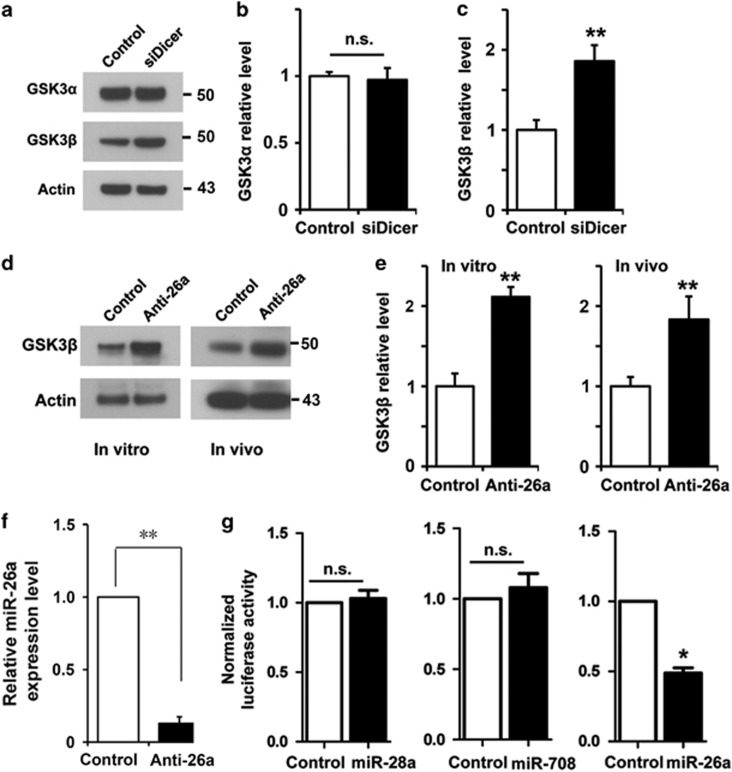
Endogenous miR-26a targets glycogen synthase kinase 3*β* (GSK3*β*) in adult mouse sensory neurons *in vitro* and *in vivo*. (**a**) Representative western blot images of GSK3*α* and GSK3*β* in adult mouse sensory neurons 3 days after Dicer knockdown. (**b**) Quantification of GSK3*α* level (normalized to actin, *n*=3). (**c**) Quantification of GSK3*β* level (normalized to actin, *n*=3). (**d**) Representative western blot images of GSK3*β* in adult mouse sensory neurons *in vitro* and *in vivo* 3 days after inhibition of miR-26a. (**e**) Quantification of GSK3*β* levels *in vitro* and *in vivo* (normalized to actin, *n*=3 for each condition). (**f**) Expression of miR-26a inhibitor in adult mouse sensory neurons markedly reduced the level of endogenous miR-26a. (**g**) Luciferase assays in neuronal cell line CAD, in which the luciferase reporter containing the full-length *GSK3β* 3′-UTR and miR-26a, miR-28a or miR-708, were coexpressed (*n*=2). Bar graphs are shown as means±S.E.M. *P<0.05 and **P<0.005; NS, not significant

To determine if GSK3s were regulated by microRNAs in adult sensory neurons, we first used siRNA to knock down Dicer, the endoribonuclease responsible for microRNA processing. We found that the level of GSK3*β*, but not GSK3*α*, was significantly increased (Figures 1a–c) upon Dicer knockdown, indicating that GSK3*β* in sensory neurons is regulated by the microRNAs. To identify the specific microRNA targeting GSK3*β*, we used a neuronal cell line to test the regulatory effects of several potential candidates based on the 3′-untranslated region (3′-UTR) sequence of GSK3*β* and previous studies, including microRNA-23b (miR-23b), microRNA-28a (miR-28a), microRNA-221 (miR-221), microRNA-135b (miR-135b), microRNA-101a (miR-101a), microRNA-26a (miR-26a) and microRNA-603 (miR-603). The results showed that miR-26a had the strongest effect specifically on GSK3*β*, but not GSK3*α* (Supplementary Figure S1). We thus examined if endogenous miR-26a regulated GSK3*β* in adult mouse sensory neurons by introducing the miR-26a inhibitor, which is a single-stranded nucleic acid designed to specifically bind and inhibit endogenous microRNAs. In our previous study,^8^ we have used these microRNA inhibitors to reduce efficiently the level of the endogenous microRNA target in adult sensory neurons. Electroporation of the miR-26a inhibitor into cultured sensory neurons led to markedly increased level of GSK3*β* (Figures 1d and e). To determine if miR-26a-regulated GSK3*β* in sensory neurons *in vivo*, we used our recently developed *in vivo* electroporation technique, which had allowed acute regulation of gene expression in dorsal root ganglion (DRG) neurons of adult mice.^9, 14^ *In vivo* electroporation of the miR-26a inhibitor into mouse DRGs also resulted in the elevated level of GSK3*β* (Figures 1d and e). To verify the effectiveness of the miR-26a inhibitor, we found that electroporation of miR-26a inhibitor into sensory neurons markedly reduced the level of the endogenous miR-26a (Figure 1f). To further confirm that miR-26a directly targeted GSK3*β*, we constructed a GSK3*β* luciferase reporter plasmid containing its full-length 3′-UTR. The miR-26a, miR-28a or miR-708 was coexpressed with the GSK3*β* 3′-UTR reporter, respectively, in a neuronal cell line CAD, which allowed high-efficiency transfection. The result showed that only overexpression of the miR-26a repressed luciferase expression (Figure 1g). This result demonstrated that miR-26a specifically repressed GSK3*β* expression through the predicted target site in its 3′-UTR. Because miR-26b shares a highly similar sequence to that of miR-26a, we also examined if miR-26b targeted GSK3*β* in adult mouse sensory neurons. To our surprise, we found that overexpression of the miR-26b inhibitor in sensory neurons had no effect on GSK3*β* expression (Supplementary Figure S1).

A previous study has shown that overexpression of the miR-26a mimics could reduce the level of phosphatase and tensin homolog (Pten) in cultured rat embryonic cortical neurons.^15^ However, we found that the levels of Pten and phosphorylated Akt in adult sensory neurons were not affected by the expression of the miR-26 inhibitor. In addition, the level of total Akt was also not affected by the miR-26a inhibitor (Supplementary Figure S2), indicating that endogenous miR-26a in adult sensory neurons does not target Pten. Taken together, these experiments provided clear and strong evidence that miR-26a acted as a physiological regulator in mouse adult sensory neurons to maintain GSK3*β* at a lower level.

### Endogenous miR-26a regulates sensory axon regeneration *in vitro* and *in vivo*

**Figure 2:**
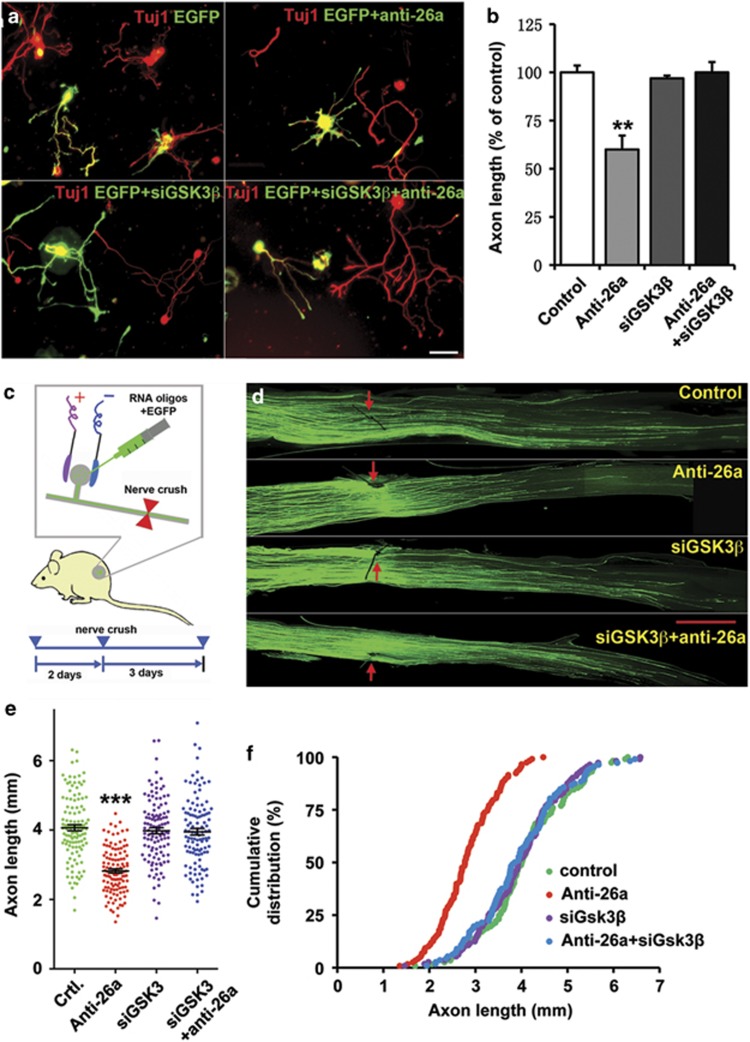
MiR-26a supports sensory axon regeneration by targeting GSK3*β* *in vitro* and *in vivo*. (**a**) Representative images of cultured adult mouse sensory neurons expressing EGFP, EGFP+miR-26a inhibitor (anti-26a), EGFP+GSK3*β* siRNA (siGSK3*β*) and EGFP+anti-26a+siGSK3*β*. All neurons were stained with anti-*β*III tubulin antibody (Tuj-1, red). Scale bar=100 *μ*m. (**b**) Quantification of the average length of the longest axons (normalized to the average length of the control axons, *n*=3). (**c**) Schematics of the protocol for *in vivo* electroporation and sciatic nerve regeneration experiments. The miR-26a inhibitor and EGFP were electroporated together into adult mouse DRGs (L4/5) *in vivo*. Two days later, the mice were subjected to a sciatic nerve crush procedure, and axon regeneration was assessed 3 days later. (**d**) Representative images of EGFP-labeled regenerating axons in the whole-mount mouse sciatic nerves. The crush sites were marked by the epineural suture (red arrows). Scale bar=1 mm. (**e**) Scatter plot of average lengths of regenerating sciatic nerve axons (*n*=6 mice for each condition). (**f**) Cumulative distribution of lengths of all individual axons measured in the sciatic nerves (*n*=123 for control, *n*=124 for anti-26a, *n*=118 for siGSK3*β* and *n*=117 for anti-26a+siGSK3*β*). Bar graphs are shown as means±S.E.M. **P<0.005 and ***P<0.001

Our previous studies^10, 16^ have shown that GSK3*β* functions to control sensory axon growth and regeneration. We thus studied the functional role of miR-26a in the regulation of sensory axon regeneration. To inhibit endogenous miR-26a and at the same time label transfected neurons, we co-transfected sensory neurons with the miR-26a inhibitor and EGFP via electroporation. The transfection efficiency through electroporation was mainly determined by the size of the plasmid. Based on our previous studies,^8, 10, 14, 17^ the transfection efficiencies of sensory neurons with EGFP (~4.5 kb) are about 30–50% in dissociated neurons and 5–10% *in vivo*. However, the transfection efficiency of small RNA oligos (e.g. siRNA, microRNA inhibitor) is much higher (>90%) based on our previous studies.^8, 10, 14^ To examine directly the transfection efficiency of microRNA inhibitor in adult sensory neurons via electroporation, we used a fluorescence dye-labeled microRNA inhibitor, miRIDIAN microRNA Hairpin Inhibitor Transfection Control with Dy547. The result showed that by electroporation 95.19±0.57% cells were labeled with the red dye after overnight culture (Supplementary Figure S3). Thus, we thought that most EGFP-positive neurons should be positive for the miR-26a inhibitor. To examine the role of miR-26a in regenerative axon growth *in vitro*, the transfected neurons were cultured for 3 days and then replated as described in our previous study.^10^ Regenerative axon growth was analyzed after overnight culture of replated neurons. The result showed that expression of the miR-26a inhibitor significantly blocked axon growth of adult sensory neurons (Figures 2a and b).

To examine the role of miR-26a in sensory axon regeneration *in vivo*, we used our recently developed *in vivo* electroporation approach, which has been used in many of our previous studies,^8, 9, 10, 14, 18^ to introduce both miR-26a inhibitor and EGFP into adult mouse DRG neurons. Two days later, the mice were subjected to a sciatic nerve crush procedure and sensory axon regeneration was assessed 3 days later. As described in our previous studies, we used the whole-mount sciatic nerves to trace the full path of every EGFP-labeled axon from the crush site (tagged with size 10-0 suture) to the axonal tip, which allowed us to measure directly the lengths of regenerating axons. The result showed that sensory neurons expressing miR-26a inhibitor and EGFP had significantly impaired axon regeneration *in vivo* compared with those of control neurons expressing EGFP alone (Figures 2c–f). Taken together, our results demonstrated that endogenous miR-26a is necessary for axon regeneration both *in vitro* and *in vivo*.

### The regulatory effect of endogenous miR-26a on sensory axon regeneration is mediated by increased level of GSK3*β*

To determine if inhibition of miR-26a impaired sensory axon regeneration via controlling the GSK3*β* level, we performed rescue experiments to examine if downregulation of GSK3*β* was able to reverse the inhibitory effect on axon growth by the miR-26a inhibitor. First, we co-transfected dissociated sensory neurons with the miR-26a inhibitor, siRNAs targeting GSK3*β* and EGFP plasmid. The siRNAs against GSK3*β* were a group of four different siRNAs designed to minimize the off-target effects (ON-TARGETplus; Thermo Scientific Dharmacon, Chicago, IL, USA). Similarly, the transfected neurons were cultured for 3 days, replated and regenerative axon growth was analyzed after overnight culture of replated neurons. We found that downregulation of endogenous GSK3*β* alone had no effect on axon growth. As shown in our recent study,^10^ the kinase activities of both GSK3*α*/*β* were markedly reduced in sensory neurons after 3 days in culture. Thus, additional downregulation of GSK3*β* had no additive effect. Importantly, downregulation of GSK3*β* fully rescued axon growth that was blocked by the miR-26a inhibitor (Figures 2a and b).

We next performed similar rescue experiments *in vivo* by coelectroporating the miR-26a inhibitor, EGFP, and/or GSK3*β* siRNAs into mouse DRGs. Two days later, the mice were subjected to a sciatic nerve crush procedure and sensory axon regeneration was assessed 3 days later. Similarly, knocking down GSK3*β* alone also had no promoting effect on sensory axon regeneration *in vivo*, but it also fully rescued sensory axon regeneration impaired by the miR-26a inhibitor (Figures 2c–f). Taken together, these results indicated that GSK3*β* was the major target of miR-26a in adult mouse sensory neurons that functioned to control axon regeneration.

### miR-26a controls axon regeneration via GSK3*β* by targeting gene expression

**Figure 3:**
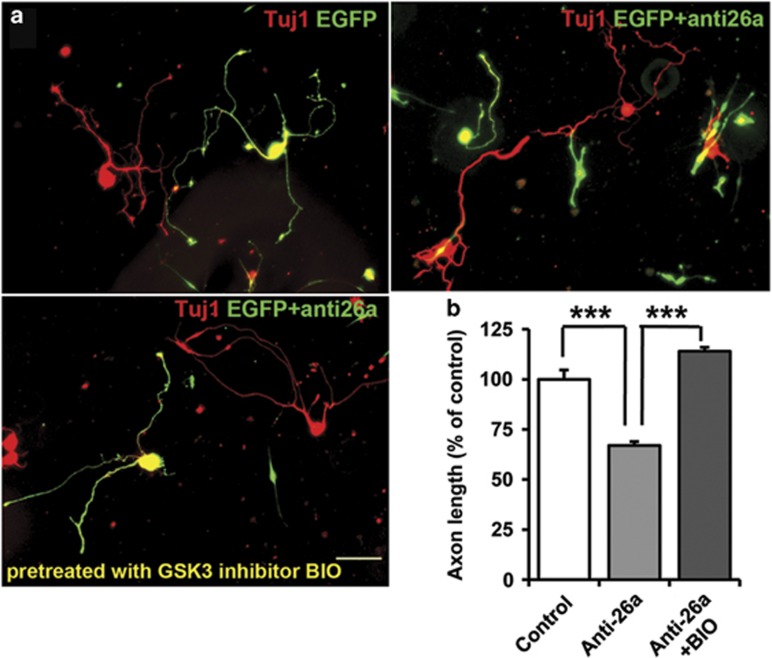
MiR-26a-GSK3*β* regulate sensory axon regeneration by controlling gene expression. (**a**) Representative images of cultured adult mouse sensory neurons expressing EGFP, EGFP+miR-26a inhibitor (anti-26a) and EGFP+anti-26a treated with the GSK3 inhibitor BIO before cell replating. All neurons were stained with anti-*β*III tubulin antibody (Tuj-1, red). Scale bar=100 *μ*m. (**b**) Quantification of the average length of the longest axons (normalized to the average length of the control axons, *n*=3). Bar graphs are shown as means±S.E.M. ***P<0.001

Our previous studies have revealed that GSK3 signaling can regulate axon growth either at the growth cone via controlling microtubule dynamics or at the cell soma via controlling gene expression.^9, 10^ In particular, we have recently established an *in vitro* model of neuronal replating,^10^ which can be used to distinguish such spatial dependent roles of GSK3 signaling. Specifically, we have shown that when sensory neurons were cultured for 3 days and replated, axon growth after replating could be affected by cytoskeletal reagents (e.g. taxol) but not transcription inhibitor. Conversely, when neurons were treated before replating, only transcription inhibitor but not cytoskeletal reagents could affect axon growth. Here we transfected sensory neurons with the miR-26a inhibitor and at the same time treated the cells with a specific GSK3 inhibitor 6-bromoindirubin-3′-acetoxime (BIO). After 3 days in culture, the neurons were replated and cultured overnight in the absence of the GSK3 inhibitor. The result showed that inhibition of GSK3 activity before cell replating completely rescued axon growth blocked by the miR-26a inhibitor (Figure 3). Because the sensory neurons still possessed functioning miR-26a inhibitor when they were replated and cultured overnight, this result suggested that miR-26a did not regulate GSK3*β* at the nerve growth cone to control microtubule assembly. We have shown before that treating cells with the GSK3 inhibitor alone could slightly promote axon growth.^10^ Therefore, the result here suggests that miR-26a control axon regeneration via GSK3*β* by targeting gene expression at the neuronal soma.

### miR-26a regulates sensory axon regeneration via regulation of Smad1 downstream of GSK3*β*

**Figure 4:**
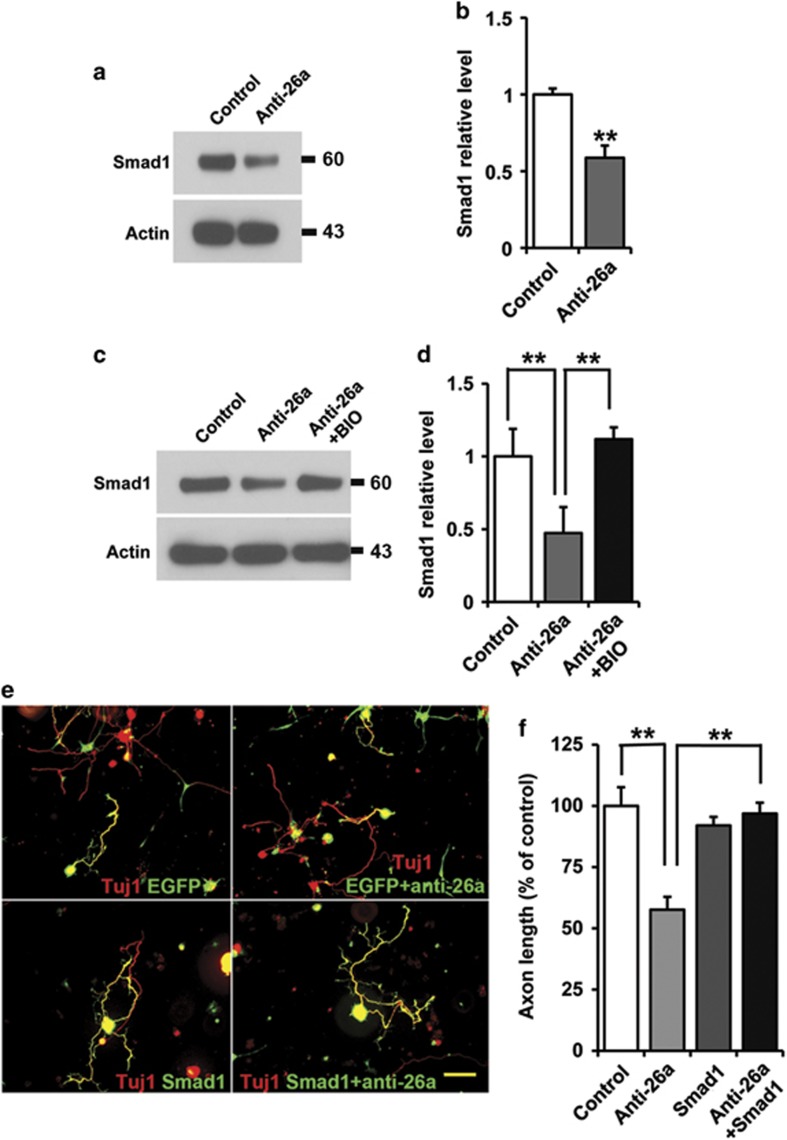
MiR-26a-GSK3*β* regulate axon regeneration by controlling Smad1 expression. (**a**) Representative western blot images of Smad1 in cultured adult mouse sensory neurons 3 days after inhibition of miR-26a. (**b**) Quantification of Smad1 level (normalized to actin, *n*=3). (**c**) Representative western blot images of Smad1 in cultured adult mouse sensory neurons 3 days after inhibition of miR-26a with or without the treatment the GSK3 inhibitor BIO. (**d**) Quantification of Smad1 level (normalized to actin, *n*=3). (**e**) Representative images of cultured adult mouse sensory neurons expressing EGFP, EGFP+miR-26a inhibitor (anti-26a), Smad1 and Smad1+anti-26a. Scale bar=100 *μ*m. (**f**) Quantification of the average length of the longest axons (normalized to the average length of the control axons, *n*=3). Bar graphs are shown as means±S.E.M. **P<0.005

To elucidate how miR-26a and GSK3*β* regulate gene expression during sensory axon regeneration, we examined the regeneration-associated transcription factor Smad1, a known downstream target of the GSK3 signaling.^10^ We found that the protein level of Smad1 was significantly decreased in adult sensory neurons upon expression of the miR-26a inhibitor (Figures 4a and b). Moreover, such reduction could be fully rescued by treating the neurons with the GSK3 inhibitor (Figures 4c and d). These results demonstrated clearly that the miR-26a-GSK3*β* pathway functioned to control the expression of Smad1 and subsequent Smad1-mediated gene expression. Functionally, when Smad1 was co-transfected into sensory neurons together with the miR-26a inhibitor, it completely rescued axon growth blocked by the miR-26a inhibitor (Figures 4e and f). Overexpression of Smad1 alone had no promoting effect on axon growth, similar to what we observed before,^10^ further confirming the rescuing effect of Smad1. Collectively, our study provided both biochemical and functional evidences to support a miR-26a-GSK3*β*-Smad1 pathway in controlling mammalian axon regeneration.

## Discussion

Efficient axon growth requires coordinated gene expression in the neuronal soma and local axon assembly at the nerve growth cone.^19^ Epigenetic modification has recently become a major mechanism in the regulation of gene expression in many biological processes in the nervous system.^20^ Our previous study has identified microRNA-138 and its target SIRT1 as important regulators of gene expression during mammalian axon regeneration.^8^ In this study, we revealed that miR-26a had an important role in the regulation of mammalian axon regeneration through its target GSK3*β* and the subsequent expression of regeneration-associated transcription factor Smad1.

Here we only identified GSK3*β*, but not GSK3*α*, as a target of microRNA regulation in adult mouse sensory neurons. One potential explanation is the much shorter 3′-UTR of the *GSK3α* gene, which might deplete its binding sites of microRNAs. The same microRNA can target multiple genes and the same gene can be targeted by multiple microRNAs, depending on the specific cellular environment. Although several previous studies in non-neuronal cells^21, 22, 23^ have shown that miR-26a targets GSK3*β*, here we demonstrated for the first time that miR-26a is a physiological regulator of GSK3*β* in adult mouse sensory neurons to control axon regeneration. Inhibition of endogenous miR-26a in adult sensory neurons led to increased level of GSK3*β* and reduced axon regeneration *in vitro* and *in vivo*. Moreover, miR-26a could directly bind to the 3′-UTR of the *GSK3β* gene in the luciferase assay. Most importantly, downregulation of GSK3*β* could fully rescue axon regeneration impaired by inhibition of miR-26a *in vitro* and *in vivo*. In addition to GSK3*β*, miR-26a could target other genes. For instance, a recent study^15^ has shown that miR-26a acts to support axon growth of embryonic rat cortical neurons by repressing Pten expression. However, in that study only overexpression of miR-26a mimics was studied and the role of endogenous miR-26a was not investigated. Indeed, another recent study that was more thoroughly performed showed that endogenous miR-17-92 functioned to control axon growth of embryonic mouse cortical neurons through targeting Pten.^24^ In a different study, miR-222 was shown to target Pten and control axon growth in adult sensory neurons.^25^ Here we showed that both Pten and phospho-Akt levels were not affected when the endogenous miR-26a was inhibited. Our results demonstrated that in adult mouse sensory neurons GSK3*β* was the major target of endogenous miR-26a in the regulation of axon regeneration.

Our previous studies^10, 17^ have shown that GSK3 kinase activity is reduced upon peripheral nerve injury, and such decreased GSK3 activity is necessary for efficient axon regeneration. In these studies, the protein level of GSK3*β* was not changed upon peripheral axotomy. However, it does not suggest that regulation of GSK3*β* protein level is not important for axon regeneration. Our study here indicates that the GSK3*β* protein needs to be maintained at a lower level by miR-26a in adult mouse sensory neurons to support efficient axon regeneration. Loss of miR-26a function led to elevated level of GSK3*β* and markedly reduced axon regeneration. Therefore, our study revealed a novel regulatory mechanism of GSK3 signaling in neurons during regeneration. In some neurological disorders, such as Alzheimer's disease, the protein level of GSK3*β* has been shown to be elevated in the brain,^26^ suggesting that such mechanism also has an important role in other neurological processes. Results from our previous studies^9, 10^ indicated that inactivation of GSK3 activity regulated axon regeneration in a coordinated way at the growth cone to control microtubule dynamics and in the neuronal soma to control gene expression. In this study, by using the cell replating experiments developed in our earlier study,^10^ we provided evidence that miR-26a mainly regulated GSK3*β* at the neuronal soma to control gene expression. In support, we identified transcription factor Smad1 as a downstream target of the miR-26a-GSK3*β* pathway.

A recent study^12^ has shown that knocking out GSK3*β* enhanced sensory axon regeneration in the spinal cord. However, we showed that downregulation of GSK3*β* had no promoting effect on sensory axon regeneration in the peripheral nerve. One likely reason for the different results is that axon regeneration over permissive environment (peripheral nerve) is mainly determined by the intrinsic axon growth ability, whereas axon regeneration over inhibitory environment (spinal cord) also requires the axons to overcome the inhibitory signals. Thus, GSK3*β* knockout could promote axon regeneration in the spinal cord by overcoming regeneration inhibitors, similar to a previous study using GSK3 inhibitors.^11^ As a result, we think that overexpression of miR-26a in sensory neurons is unlikely to promote peripheral nerve regeneration, but whether it could promote axon regeneration in the CNS is an interesting question to be explored in the future.

In summary, in this study we identified miR-26a as an important regulator of mammalian axon regeneration *in vivo*. We provide the first evidence that, in addition to inhibition of GSK3*β* kinase activity, maintaining lower protein level of GSK3*β* in neurons by the microRNA is necessary for efficient axon regeneration. Lastly, although GSK3*β* and Smad1 have been shown to be involved in axon regeneration, our study revealed miR-26a as a novel upstream regulator of the GSK3*β*-Smad1 pathway.

## Materials and Methods

### Animals

All animals used in the experiments were handled according to the guidelines of the Institutional Animal Care and Use Committee of the Johns Hopkins University. The 8- to 12-week-old adult female CF-1 mice (weighing from 30 to 35 g) were purchased from Charles River Laboratories (Wilmington, MA, USA) and housed in the University Animal Facility.

### Reagents and antibodies

The mouse mmu-miR-26a-5p-inhibitor, miRIDIAN microRNA Hairpin Inhibitor Transfection Control with Dy547, siRNAs against mouse GSK3*β* and Dicer (ON-TARGETplus SMART POOL), were purchased from Thermo Scientific Dharmacon. The GSK3*β* antibody was from BD Biosciences (Franklin Lakes, NJ, USA). The actin antibody was from Sigma-Aldrich (St. Louis, MO, USA). The *β*III tubulin antibody (TuJ1) was from Covance (Chantilly, VA, USA). Antibodies against GSK3*α*, phospho-GSK3*β*, Smad1 and Pten were from Cell Signaling Technology (Beverly, MA, USA). The pCMV-GFP and pIS2 plasmids were from Addgene (Cambridge, MA, USA). All fluorescence-tagged secondary antibodies were from Molecular Probes Inc. (Eugene, OR, USA). BIO was from Calbiochem (San Diego, CA, USA).

### Primary culture of adult mouse sensory neurons

The adult mouse sensory neurons were cultured using the same protocol as our previous studies.^8, 10^ Briefly, DRGs from adult mice were dissected out and digested at 37 °C with collagenase A (1 mg/ml) for 90 min, followed by treatment with trypsin-EDTA (0.05%) for 20 min. The DRGs were washed three times with the culture medium (MEM with l-glutamine, penicillin/streptomycin and 5% fetal calf serum), and then dissociated with a 1 ml pipette tip in the culture medium. The dissociated neurons were centrifuged down and plated onto 24- or 12-well plates coated with a mixture of 100 *μ*g/ml poly-d-lysine and 10 *μ*g/ml laminin. The plated neurons were cultured in the culture medium described above in the absence of any added growth factors. Three days later, the neurons were resuspended and replated onto glass coverslips coated with a mixture of 100 *μ*g/ml poly-d-lysine and 10 *μ*g/ml laminin. Replated adult sensory neurons were fixed with 4% paraformaldehyde after overnight culture.

To transfect RNA oligos and/or DNA plasmids into DRG neurons, the dissociated neurons were centrifuged to remove the supernatant and resuspended in 80–100 *μ*l of Amaxa electroporation buffer (for mouse neuron; Lonza Cologne GmbH, Cologne, Germany) with siRNAs or microRNA inhibitor (0.2 nmol per transfection) and/or the EGFP (10 *μ*g per transfection) plasmid. Suspended cells were then transferred to a 2.0-mm cuvette and electroporated with the Amaxa Nucleofector apparatus. After electroporation, cells were immediately mixed to the desired volume of prewarmed culture medium and plated on culture dishes. After neurons were fully attached to the coverslips (4–6 h), the culture medium was changed to remove the remnant electroporation buffer.

### *In vivo* electroporation of adult mouse DRGs and sciatic nerve crush

The procedure was the same as described in our previous published papers.^14^ Briefly, the L4 and L5 DRGs on one side of adult mice were surgically exposed after mice were anaesthetized. Solution containing siRNAs and/or EGFP plasmid (1 *μ*l) was slowly injected into the DRG using the capillary pipette powered by the Picospritzer II (Parker Inc., Cleveland, OH, USA; pressure: 30 psi; duration: 8 ms). The electroporation was performed immediately using a custom-made tweezer-like electrode (Ø1.2 mm) and an ECM830 Electro Square Porator BTX (five 15 ms pulses at 35 V with 950 ms interval). The incision sites were then closed, and the mice were allowed to recover after surgery. For peripheral nerve regeneration experiments, the sciatic nerve on the side of the electroporated DRGs was crushed 2 days later and the crush site was marked with a size 10-0 nylon epineural suture. After surgeries, the wound was closed and the mice were allowed to recover. Three days after nerve crush, the mice were terminally anesthetized and transcardially perfused with PBS (pH 7.4), followed with ice-cold 4% paraformaldehyde in PBS. The whole sciatic nerve were dissected out and further fixed in 4% paraformaldehyde overnight at 4 °C. Before whole-mount flattening, the place of epineural suture was confirmed to match the injury site, and experiments were included in the analysis only when the crush site was clearly identifiable.

### Measurement of axon growth *in vitro* and axon regeneration *in vivo*

For measurement of axon growth *in vitro*, the fixed neurons were washed with PBS and blocked in blocking buffer (2% BSA, 0.1% Triton X-100, 0.1% sodium azide in PBS) for 1 h. The neurons were then immunostained with the antibody against neuronal *β*III tubulin (Tuj-1). The fluorescence images of neurons were captured with an inverted fluorescence microscope controlled by the Axio Vision 4.6 software (Carl Zeiss MicroImaging Inc., Oberkochen, Germany). Only neurons with axons longer than two times the diameter of cell bodies were selected. The longest axon of each neuron was traced manually and measured. In each experiment, 50–100 neurons per condition were measured, and at least three independent experiments were performed to calculate the mean value of axon length. The '*n*' in the figures indicates the number of independent experiments.

For quantification of axon regeneration *in vivo*, the fluorescence images of the whole-mount nerves were acquired using the MosaiX module of the AxioVision software (Carl Zeiss Microimaging Inc.). All identifiable EGFP-labeled axons in the sciatic nerve were manually traced from the crush site to the distal axonal tips to measure the length of the regenerated axons. Only nerves with at least 15 identifiable axons were measured and data from six mice for each experimental condition were used to calculate the mean axon length.

### Western blot analysis

DRG tissues or dissociated DRG neurons were collected and lysed using the RIPA buffer. The extracted proteins were separated by 4–12% gradient SDS-PAGE gel electrophoresis, and transferred onto polyvinylidene fluoride membranes. After blocking with 10% non-fat milk, membranes were incubated with primary antibodies (4 °C, overnight), followed by HRP-linked secondary antibodies (room temperature, 1 h). The densities of protein bands from three independent experiments were quantified using the Image J software (NIH, Bethesda, MD, USA). The artificial unit of each protein was calculated by normalizing the band of the protein of interest to the band of loading control, actin.

### Quantification of mature microRNA

The level of miR-26a in *in vitro* cultured DRG neurons was tested with qRT-PCR 3 days after miR-26a inhibitor transfection. The experiment was conducted following the procedure described by a previous study.^27^ Briefly, total RNA was isolated with the TRizol reagent (Invitrogen), thereafter miR-26a and RNU6B were reverse transcribed using sequence-specific primers and Moloney murine leukemia virus reverse transcriptase (Roche Applied Science, Indianapolis, IN, USA). To quantify the level of miR-26a with the RT-PCR, aliquots of single-stranded cDNA were amplified with gene-specific primers and LightCycler 480 SYBR Green I Master (Roche, Indianapolis, IN, USA) using the LightCycler 480 detection system (Roche). The PCR reactions contained 20 ng of cDNA, Master Mix (Roche), and 200 nM forward and reverse primers in a final reaction volume of 20 *μ*l. Each assay was carried out in triplicate for each sample tested. Relative quantities of miR-26a were calculated using the DDCT method with RNU6B as the endogenous control and calibrated to the control samples. The sequences of the miR-26a primers used were sequence-specific reverse transcription, 5′-GTCGTATCCAGTGCAGGGTCCGAGGTATTCGCACTGGATACGACAGCCTAT-3′ forward, 5′-GAGTGTTTCAAGTAATCCAGG-3′ and reverse, 5′-GCAGGGTCCGAGGTATTC-3′. The sequences of the RNU6B primers used were forward, 5′-CTCGCTTCGGCAGCACA-3′ reverse, 5′-AACGCTTCACGAATTTGCGT-3′ the sequence-specific reverse transcription primer of RNU6B was the same as its reverse PCR primer.

### GSK3*β* 3′-UTR dual-luciferase assay

The assay was similar to that described in our previous study.^8^ Briefly, mouse cDNA was used to clone 3′-UTR of *GSK3β*. The sequences of the primers used were: forward, 5′-TCGCTCGAGCCAGCACTTACTTGAGTGCCAC-3′ and reverse, 5′-CCAGCGGCCGCAGCTGGAAGCTGCACCTCCTGTC-3′. The primers were designed to include *Xho*I and *Not*I sites. The PCR product digested with *Xho*I and *Not*I was inserted into the pIS2 vector to generate a luciferase 3′-UTR reporter construct. Luciferase expression was detected using the dual-luciferase reporter 1000 system (Promega) according to the manufacturer's protocol.

### Statistics

Data are presented as mean±S.E.M. Two-tailed Student's *t*-test was used to determine the statistical significance between different experimental groups, which was set at a value of *P*<0.05.

## Abbreviations

miR: microRNA
GSK3: glycogen synthase kinase 3
CNS: central nervous system
DRG: dorsal root ganglion
3′-UTR: 3′-untranslated region
Pten: phosphatase and tensin homolog
