# Supplementary material for: MicroRNA-26a supports mammalian axon regeneration in vivo by suppressing GSK3β expression
Source: Cell Death Dis. 2015 Aug 27;6(8):e1865–. doi: 10.1038/cddis.2015.239 (PMC4558520; doi:10.1038/cddis.2015.239)
Supplement: Supplementary Information [file cddis2015239x1.pdf]

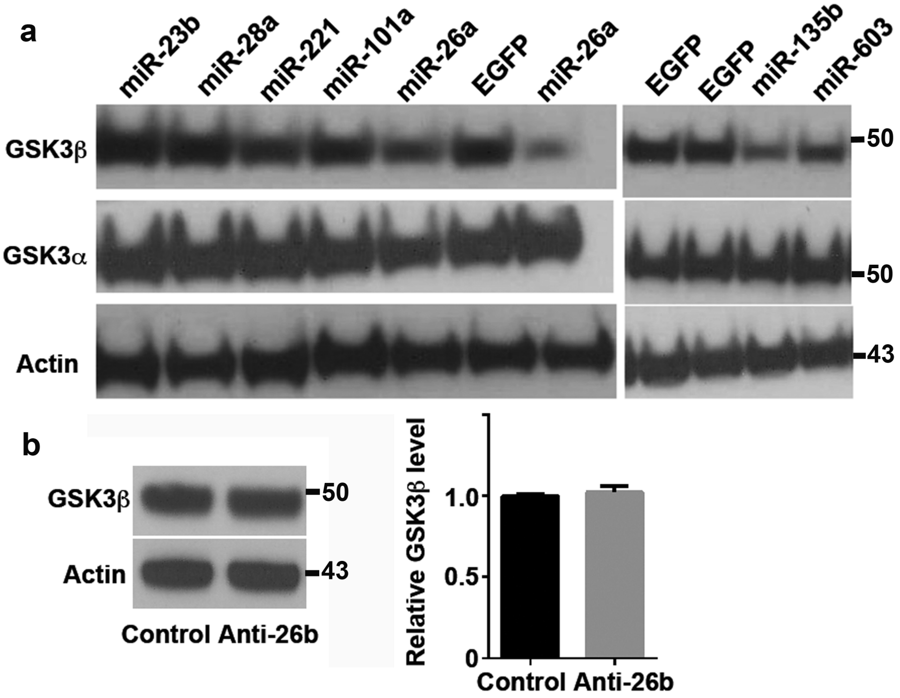


**Supplementary Figure S1. miR-26a targets GSK3β but not GSK3α. (a**) In a neuronal cell line miR-26a most efficiently regulated GSK3β expression. (**b**) In adult mouse sensory neurons, miR-26b had no regulatory effect on GSK3β expression.

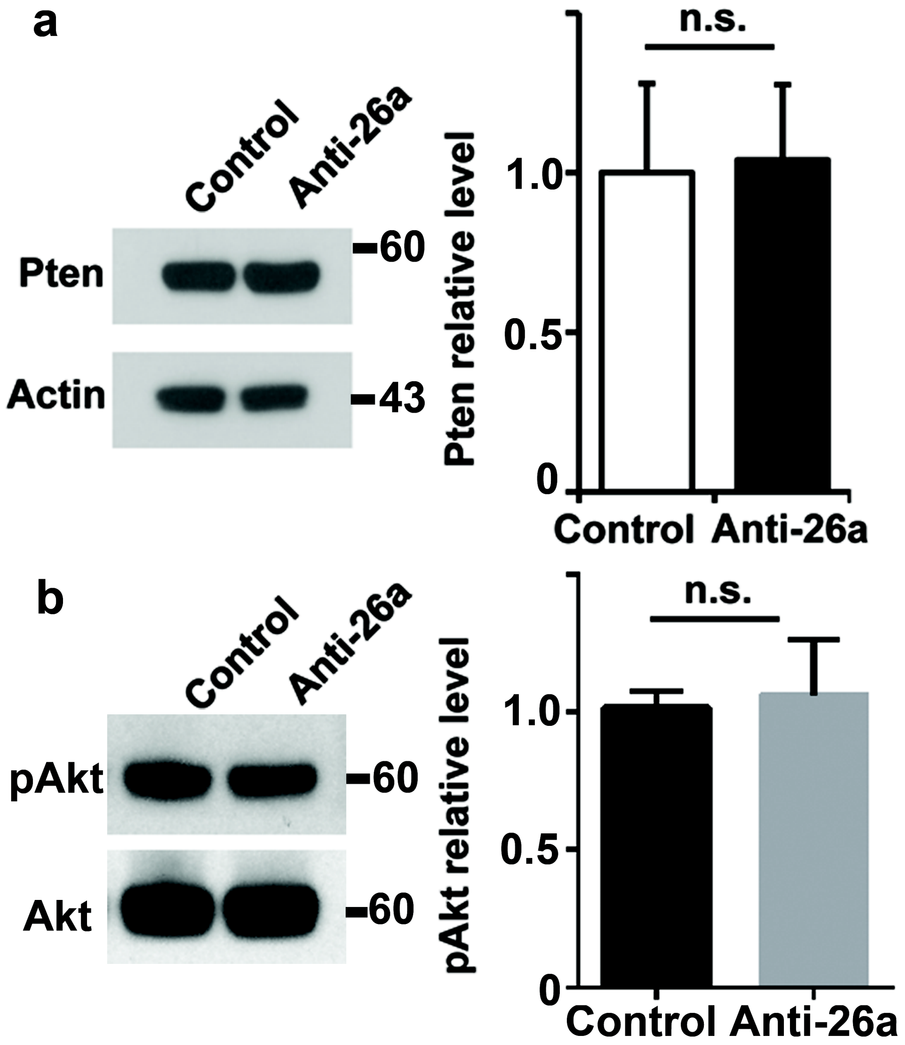


**Supplementary Figure S2. Endogenous miR-26a does not target Pten pathway in adult mouse sensory neurons.** (**a**) Representative western blot image and quantification of Pten (normalized to actin, n = 3) in cultured adult mouse sensory neurons 3 days after inhibition of miR-26a (anti-26a). (**b**) Representative western blot image and quantification of phosphorylated Akt (normalized to Akt, n = 3) in cultured adult mouse sensory neurons 3 days after inhibition of miR-26a.

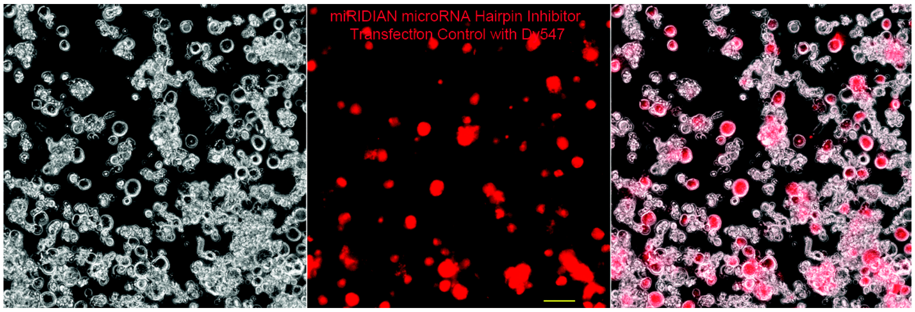


**Supplementary Figure S3. Electroporation of fluorescence dye labeled microRNA inhibitor in adult mouse sensory neurons.** Note the high transfection efficiency. Scale bar = 100 μm.
